# How Does Psychological Empowerment Prevent Emotional Exhaustion? Psychological Safety and Organizational Embeddedness as Mediators

**DOI:** 10.3389/fpsyg.2021.546687

**Published:** 2021-07-19

**Authors:** Hao Zhou, Jingyi Chen

**Affiliations:** Business School, Sichuan University, Chengdu, China

**Keywords:** psychological empowerment, emotional exhaustion, psychological safety, organizational embeddedness, conservation of resources theory

## Abstract

Emotional exhaustion in the workplace can cause employees psychological and physical health problems, affect work performance, and create burdens for the organization. Existing studies have demonstrated that psychological empowerment helps reduce emotional exhaustion. This study explores the internal mechanism of this relationship. Drawing on conservation of resources theory, we advance a dual mediation model to explain how high psychological empowerment results in low emotional exhaustion, by increasing psychological safety and organizational embeddedness. Data were collected from 226 on-the-job MBA students at a university in western China. The results demonstrate that psychological safety and organizational embeddedness play mediating roles in the negative relationship between psychological empowerment and emotional exhaustion. The study provides a systematic view of the negative effect that psychological empowerment has on emotional exhaustion. The paper also discusses theoretical contributions, practical implications, and future directions.

## Introduction

Burnout is an important emerging risk impacting career prospects and poses a major challenge for occupational safety and health in organizations (Ayala Calvo and García, 2018). Burnout is a comprehensive manifestation of emotional exhaustion, decreased personal accomplishment, and depersonalization (Maslach et al., 2001). Emotional exhaustion captures the key strain dimension of burnout and occurs when one is emotionally drained because of contact with others (Bakker et al., 2014). This includes the draining of personal resources, together with the feeling that one no longer has the ability to provide anything psychologically to others (Ayala Calvo and García, 2018). Cordes and Dougherty (1993) argued that the key determinants of emotional exhaustion reflect the demands that organizations and individuals place on employees. In other words, emotional exhaustion is unlikely to occur when people have sufficient resources to cope with daily work demands.

Emotional exhaustion has consistently been a theme of interest for scholars, because it significantly impacts quality of work life and the optimization of organizational functions. Emotional exhaustion affects job performance, health, citizenship behavior, and voluntary turnover (Wright and Cropanzano, 1998; Cropanzano et al., 2003; Halbesleben and Buckley, 2004). This highlights the importance of effectively managing emotional exhaustion. Previous studies show that predictors of emotional exhaustion include both situational and personal aspects (Zheng et al., 2015). Conservation of resources theory offers particularly valuable insights to the study of prolonged emotional exhaustion (Wright and Cropanzano, 1998).

Many studies have applied COR theory to explore what resources (e.g., leadership, social support, and resilience) affect emotional exhaustion. Further, many scholars use psychological empowerment to explain the mechanism by which these resources are transformed into an individual’s ability to cope with emotional exhaustion (Boudrias et al., 2012; Tian et al., 2015; Ayala Calvo and García, 2018; Liu et al., 2019; Permarupan et al., 2020). Previous studies found that highly empowered employees benefitted more from resources associated with organizational change compared to their counterparts, lowering risks of emotional exhaustion (Tian et al., 2015). However, past studies have noted that the mechanisms of how psychological empowerment prohibit emotional exhaustion are not well understood (Quiñones et al., 2013). Exploring this question is important for understanding how to truly transform different resources into forms that employees can use to cope with different factors related to emotional exhaustion at work. As such, we focused our research on the intermediate mechanism between psychological empowerment and emotional exhaustion.

As previously mentioned, emotional exhaustion actually reflects a state in which work resources cannot meet work demands. According to Maslow’s hierarchy of needs, people’s need for safety is one of the most basic kind. Empowered employees perceive having more resources (Gist, 1987), and we notice the psychological safety. Psychological safety describes how people think about the possible risks and consequences of interpersonal communication they face in a specific environment, such as a workplace (Edmondson and Lei, 2014). It is an essential factor which makes people feel safe and have the ability to change their behavior to face up with shifting organizational challenges (Edmondson and Lei, 2014). The loss spiral referenced in COR theory states that investment becomes more difficult when individuals lose resources (Hobfoll, 2001), such as in a changing environment. The loss of resources brings stress, leading to an iterative spiral. During this stress iteration process, individuals and organizations will lack the resources to effectively accommodate the further loss of resources, causing the momentum and amplitude of the loss spirals to rapidly increase (Hobfoll et al., 2018). This eventually leads to emotional exhaustion. But psychological safety, acting as a positive resource, helps people overcome the anxiety and defensiveness. Through the enhancement of psychological safety, psychological empowerment can prevent resource loss spirals. This alleviates the employee’s emotional exhaustion. Therefore, we hypothesized that psychological safety acts as mediator in a negative relationship between psychological empowerment and emotional exhaustion.

Organizational embeddedness describes the degree to which an employee becomes “stuck” or “enmeshed” in an organization (Lee et al., 2014). It is about fit with job and links in the organization. Research shows that in order to better counteract resource loss and promote resource appreciation, people often make resource investment based on two aspects, one is job related (e.g., job involvement and motivation) and the other is interpersonal related (e.g., trust and reciprocity; Halbesleben et al., 2014). From this perspective, we infer that organizational embeddedness can significantly help resources gaining and inhibit emotional exhaustion. Psychological empowerment helps employees perceive more links to other people or activities and feel there is a fit between other aspects of their life and the work context. The more links they feel, the more embedded they are. Consistent with the resource gain spiral of the COR theory, those with resources can more easily access and invest in additional resources (Halbesleben et al., 2014). Thus, more embedding can, in turn, help employees gain more value-added resources, helping them cope with emotional exhaustion at work. As such, we hypothesized that organizational embeddedness negatively mediates the relationship between psychological empowerment and emotional exhaustion.

The paper contributes to the current literature in three important aspects. First, we propose and validate a parallel intermediary model that interprets the underlying mechanisms of how psychological empowerment affects emotional exhaustion. Second, this study’s focus enriches the current empowerment and emotional exhaustion literature. Thirdly, the study further validates the two spirals associated with COR theory and verifies that a resource (or factor) can affect the same variable from two paths (both the resource gain and loss spirals).

In addition, these contributions demonstrate the important role of psychological empowerment in helping employees cope with emotional exhaustion. This is of great significance in the workplace and highlights that managers should further consider ways to improve psychological empowerment. Meanwhile, studying this internal mechanism shows that psychological safety and organizational embeddedness directly impact emotional exhaustion. This provides positive intervention measures in management practice.

## Literature Review and Hypotheses

### Psychological Empowerment and Emotional Exhaustion

Psychological empowerment is the manifestation of internal motivation with respect to four cognitive factors: meaning, competence, self-determination, and impact (Spreitzer, 1995). ***Meaning*** represents how well employees’ values and beliefs fit with job demands (Spreitzer, 1995). ***Competence*** (self-efficacy) reflects how confident individuals are that their skills will help them succeed in the job (Bandura, 1989). ***Impact*** describes the extent to which individuals believe their abilities can affect work activities and outcomes (Ashforth, 1989). ***Self-determination*** reflects the individual’s perception of the choice to autonomously initiate and regulate work processes (Deci et al., 1989).

In summary, the four cognitive factors reflect an active orientation and feeling of control toward work. Psychological empowerment theory holds that empowered employees have a more positive orientation toward his or her work. This reflects an orientation where employees want to have, and actually have, confidence in shaping their work role and context (Spreitzer, 1995). Therefore, experiencing empowerment and intrinsic motivation can result in positive forms of work performance.

Many studies (McVicar, 2003; Laschinger et al., 2006; ÇAVUŞ and Demir, 2010; Gong et al., 2021) have demonstrated that psychological empowerment can decrease the detrimental influence of work-related stressors on burnout, acting as a potentially protective factor (Tian et al., 2015). As noted above, emotional exhaustion is a central dimension of burnout, given its relevance to physical and psychological depletion (Shirom, 1989). Emotional exhaustion is a mental health issue, which can impose significant personal and financial burdens for individuals, organizations, and society (Schermuly and Meyer, 2016). Therefore, it is essential to identify the cause of these problems and possible remedies (Maslach and Leiter, 2008). This study focused on this topic, exploring how psychological empowerment prevents emotional exhaustion.

Specifically, ***meaning*** describes the fit between the demands of working roles and one’s values, beliefs, and behaviors (Nord et al., 1990). Previous studies have highlighted that incongruities between the job and the person are early predictors of emotional exhaustion (Maslach and Leiter, 2008). Experiencing a sense of meaning implies finding a profound goal at work; it transcends the external outcomes and constitutes a foundational motivation of humanity (Arnold et al., 2007). This reduces the possibility of emotional exhaustion.

***Competence*** means self-efficacy, or the belief in the capacity to carry through tasks successfully and perform responsibilities (Bandura, 1993). Competence leads to persistence and efforts in challenging situations, the skills to cope, high target expectations, and high performance (Spreitzer, 1995). Meta-analyses have confirmed the direct relationship between self-efficacy (competence) and burnout (Aloe et al., 2014). Also, many empirical studies have demonstrated that self-efficacy is negatively relative to emotional exhaustion. Examples of this relationship include the buffering effect (Schwarzer and Hallum, 2008); the mediating role through stress as an indirect predictor of strain (Wang et al., 2015); and the antecedent effect (Rubio et al., 2015).

***Impact*** is related to the ability to get out of difficult situations and to exhibit high performance (Ashforth, 1990). Empowered people with high degree of impact believe they can affect the unit’s or organization’s strategic direction, operational processes, and outcomes (Ashforth, 1989). As a result, they have more confidence to tackle challenging tasks and can cope with stress more positively at work.

***Self-determination*** embodies autonomy and a sense of control in initiating work behaviors and continuing work processes (Deci et al., 1989). For example, this could include decisions about working methods, procedures, and efforts (Spector, 1986; Bell and Staw, 1989). Autonomy means that individuals can choose and organize actions using their own initiative (Ayala Calvo and García, 2018). This is a significant mechanism for reducing tension and stress (Spreitzer, 1997). In difficult and threatening situations, feelings of control offer employees positive, cognitive, affective, and motivational resources (Schermuly and Meyer, 2016), buffering against emotional exhaustion (Grandey et al., 2005). Maslach and Leiter (2008) found that participating in organizational decision making is negatively related to emotional exhaustion, which is one specific form of control.

Together, the four cognitive factors associated with psychological empowerment inhibit emotional exhaustion. Therefore, we hypothesized that

*H1*: Psychological empowerment is negatively related to emotional exhaustion.

### COR Theory

As a widely adopted explanatory mechanism to understand burnout and stress (Hobfoll, 2011), COR theory assumes that people usually strive to acquire, protect, retain, and cultivate the resources they think are valuable (Hobfoll et al., 2018). According to COR theory, there are four basic principles. The first is *Primacy of loss principle*. It suggests that the impact of resource loss is greater than resource gain. The second is *Resource investment principle*, which indicates that individuals must invest in resources to prevent the loss of resources, recover from loss, and obtain more resources. The third is *Gain paradox principle*. It is pointed out that when people are in the situation of resource loss, resource gains are more valuable and therefore more important. The fourth principle is *Desperation principle*. It points out that when individuals run out of resources, they will enter a defensive, aggressive, and even irrational mode to protect themselves.

In addition, COR theory puts forward three corollaries. *Corollary 1* indicates that the more resources there are, the smaller the loss of resources is, and the stronger the ability to obtain resources is. *Corollary 2: Resource loss cycles*, which suggest that resource loss has a spiral nature. Similarly, *Corollary 3: Resource gain cycles*, which suggest that resource acquisition also has a spiral nature. The three corollaries make some specific and complex predictions, which will help researchers to develop complex strategies to deal with the primary stress states at the individual or organizational level (Hobfoll et al., 2018).

When people experience the loss of resources, feel the threat of loss of resources, and are in the situation that personal resources are insufficient to meet the needs of the work, or when the investment of resources does not produce the expected return, they are more prone to emotional exhaustion (Hobfoll, 1989). Based on the resources loss and gain spirals, this study will explore the internal mechanism of the impact of psychological empowerment on emotional exhaustion.

### Psychological Safety as Mediator

Psychological safety describes how people perceive taking on the risk of interpersonal relationships in specific contexts, such as the workplace. Psychological safety is critical for individuals to feel secure and to be able to change their behaviors when facing shifting organizational challenges (Edmondson and Lei, 2014). This safety also gives individuals the freedom to concentrate on work goals and problem prevention, rather than self-protection (Schein, 1993).

The four cognitions of psychological empowerment introduced and defined above are all closely associated with improving psychological safety, as follows. Individuals with a high sense of ***competence*** believe they are more competent with work tasks and worry less about the consequences of their actions in working and interacting with colleagues. As such, they enjoy a high level of psychological safety during working activities or interactions with colleagues. ***Meaning*** gives rise to a high level of commitment and concentrates energy (Spreitzer, 1995). A higher energy focus means that individuals may experience fewer distractions (e.g., thinking about work relationships while at home) and may solve problems at work more effectively. Moreover, ***self-determination*** represents an individual’s feeling of sufficient autonomy at work (Boudrias et al., 2012). ***Impact*** is concerned with having the confidence to significantly contribute to the organization’s outcomes (Ayala Calvo and García, 2018). Employees with high levels of self-determination and impact believe they can independently grasp and arrange matters at work, and they consider their actions and activities meaningful. They do not worry significantly about whether their actions will be punished by superiors or will affect their interpersonal relationships. As a result, they experience a state of high psychological safety. Overall, psychological empowerment has positive effects on perceived psychological safety.

Conservation of resources theory holds that people with inadequate resources suffer more easily from resource loss, and the initial losses lead to future losses (Hobfoll, 2001). Because of lacking resources to mitigate the loss, loss spirals develop. If resources are used to prevent the loss of other resources, those losses will further reduce the possibility of maintaining essential resource reserves (Hobfoll, 1989).

Individuals with low psychological safety are more likely to suffer a resource loss spiral: The resources they have invested are not returned as expected. Stress resulted from lacking psychological safety could demand employees to draw on other internal resources for coping, which will induce resources loss spirals and exhaustion (Swendiman et al., 2019) Specifically, they may feel unsafe in the workplace and less confident about their ability to cope with potential risks. The lack of psychological safety may make employees more reluctant to speak or do something that could improve their initiative (Simonet et al., 2015). Then, to avoid or reduce interpersonal risks, individuals with low levels of psychological safety are more cautious in the workplace. When communicating with leaders, they may be afraid of being punished for saying or doing the wrong thing, requiring more energy and time. When communicating with colleagues, they consistently doubt other’s motives and the purpose driving others’ behaviors. This leads to feelings of suspicion, and the perceived need for more caution.

In this “self-protection” process, large amounts of resources (such as time and energy) are invested in these defensive interactions. These investments may be invalid, non-value-added, and may lead to negative effects. For example, excessive suspicion when working with others will negatively affect harmonious interpersonal relationships, hindering the development of companionship, and reducing co-worker support. This hampers good resources that could have been obtained from positive social interactions. This is consistent with the resource loss spiral of COR. The evidence above suggests that when individuals perceive lower psychological safety, the greater the possibility there will be emotional exhaustion. Conversely, employees with higher the psychological safety do not need to focus on “self-protection,” which consumes resources and eventually leads to emotional exhaustion (Rathert et al., 2020), that is to say, the less likely there is to be a loss spiral and the less likely emotional exhaustion will occur.

In summary, employees who are highly empowered perceive more psychological safety in the organizational context. They do not feel the need to invest resources in self-protection or other processes to reduce resource loss. This prevents them from falling into a loss spiral and prevents emotional exhaustion. From the perspective of a spiral in resource loss, psychological safety explains a possible path for psychological empowerment to be able to impact emotional exhaustion. Therefore, we hypothesized that

*H2*: Psychological safety mediates the negative relationship between psychological empowerment and emotional exhaustion.

### Organizational Embeddedness as Mediator

Organizational embeddedness is comprised of three interrelated dimensions: links, fit, and sacrifice (Mitchell et al., 2001). Fit refers to the degree to which a job resembles or fits with the other aspects of life. Links refer to the degree to which individuals have links to other people and activities. Sacrifice refers to whether an individual can easily break links: What they would give up if they left their present setting. Together, these three dimensions represent both attachment and inertia, because there is less likelihood that embedded employees will leave their current organizations (Peltokorpi et al., 2015). Organizational embeddedness is one sub-dimension of job embeddedness. The other sub-dimension is community embeddedness, which describes how fit, links, and sacrifice relate to community life. Together, they keep individuals in these locations (Ng and Feldman, 2014).

Psychological empowerment significantly enhances an employee’s organizational embeddedness. In terms of fit, empowered employees perceive more meaning in work; they have a strong sense that their personal values and beliefs are consistent with work demands. Mitchell et al. (2001) posit that people will be more closely tied to the organization if their personal values, career aims, and future planning efforts are a better fit with the larger organizational culture and current job demands (such as job knowledge, skills, and abilities). Therefore, the empowered people will experience more congruence with their organizational roles, which reinforced their sense of embeddedness.

At the same time, employees with high levels of psychological empowerment experience autonomy. This is reflected when they initiate work actions and continue work processes (Bell and Staw, 1989), motivating them to stay. In addition, because of an enhanced feeling of competence, empowered individuals have faith that their efforts can yield good outcomes (Bandura, 1993). This leads to a willingness to participate in work activities, creating new links with the organization and co-workers. When there is a high degree of self-efficacy and impact, people believe their roles are important. This gives them confidence in the abilities to affect job and work in meaningful ways. In these processes, employees develop more connections with their colleagues and supervisors, deepening the links they have to other people or activities.

Conservation of resources theory’s fundamental tenet states that people strive to obtain, retain, protect, and foster the things they value (Hobfoll, 2001). Greater fits and stronger links resulting from psychological empowerment are valuable resources that individuals do not want to sacrifice. Given this, we hypothesize that psychological empowerment enhances organizational embeddedness.

Conservation of resources theory notes that with a resource gain spiral, people who hold resources have more ability to gain more of them and that the initial gains generate further gains (Hobfoll, 2001). As such, the initial resource gain allows people to better invest and obtain additional resources (Halbesleben et al., 2014).

Consistent with the gain spiral of COR, embedded individuals demonstrate better fits and more links. These can serve as value-added resources that may yield more resources. Highly embedded employees are highly associated with their organizations and have high expectations with respect to future interactions with individuals or teams within the organization (Sekiguchi et al., 2008). This means that embedded employees may gain more positive resources, such as social support from co-workers or organizations, companionship, social relations, and loyalty from friends during workplace social interactions. These resources help prevent emotional exhaustion. This is because emotional exhaustion occurs when there is a lack of emotional resources to cope with stress that individuals confront (Lee and Ashforth, 1996). People share emotions and feelings through positive social interactions, healing emotional loss, and replenishing emotional resources (Heaphy and Dutton, 2008). When there are sufficient resources to address daily work demands, individuals suffer less emotional exhaustion.

Based upon the evidence above, employees with a higher sense of psychological empowerment are more embedded. They have more links with organizations and co-workers, enjoy better fits between jobs and personal values, and less likely to perceive the need to sacrifice. They have an adequate supply of resources, enjoy the resource gain spirals, and will be less threatened by the loss of resources. The resources they have can effectively meet work demands; this makes them less likely to experience emotional exhaustion. Thus, based on the resource gain spiral, organizational embeddedness explains a possible path for psychological empowerment to affect emotional exhaustion. The third study hypothesis was

*H3*: Organizational embeddedness mediates the negative relationship between psychological empowerment and emotional exhaustion.

**Figure 1:**
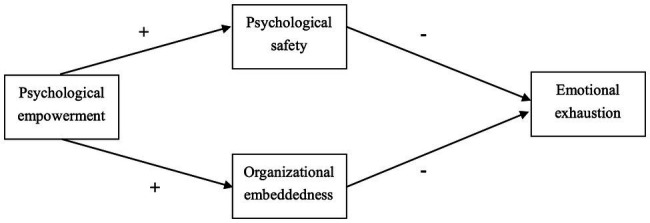
Hypothesized theoretical model.

To investigate two mediation paths based on the two resources spirals of the COR theory, we proposed a double-mediated model to explain the influence of psychological empowerment on emotional exhaustion, as shown in Figure 1.

## Materials and Methods

### Participants and Procedures

Participants were on-the-job MBA students at a university in western China. Limited by the research resources, this study adopts a convenience sampling method and only collects data in the MBA project of one school. However, the MBA students of this project come from companies with different management styles, different industries and even from various regions. Compared with data collection in one or a limited number of companies, the samples in this study can ensure that the variance of each important variable is large enough, which also assures that the results are more in line with the real situation.

For controlling common method biases, we carried out three-wave surveys, which involved three separate surveys, conducted at two-week intervals. Participants reported as: psychological empowerment, demographic variables, and zhongyong at time 1; psychological safety and organizational embeddedness at time 2; and emotional exhaustion at time 3.

This is an anonymous survey; however, respondents were required to submit their cell phone numbers every time they filled in the questionnaire. The number served as a label to match the three questionnaires and was also used to reward the respondent with a telephone credit of 10-yuan RMB after each response. To increase participant continued interest, if the three surveys matched exactly, an additional 10-yuan RMB telephone fee were awarded.

We initially issued 409 questionnaires; 226 valid questionnaires were obtained after matching and excluding invalid questionnaires (response rate = 55.26%). Demographically, the average age of participants was 32.195 years (*SD* = 5.220); 52.7% were male; and they had worked at their companies for an average 6.089 years (*SD* = 4.959).

### Measures

All measures were translated into Chinese, using a translation and back-translation procedure proposed by Brislin (1980). All scale items for this survey were assessed using 5-point Likert-format scales, with responses ranging from 1 (strongly disagree) to 5 (strongly agree).

To measure **psychological empowerment**, we used Spreitzer’s (1995) 12-item instrument, including four sub-dimensions: meaning (e.g., The work I do is very important to me), competence (e.g., I am confident about my ability to do my job), self-determination (e.g., I have considerable opportunity for independence and freedom in how I do my job), and impact (e.g., I have a great deal of control over what happens in my department). The coefficient alpha was 0.853 for this survey.

**Emotional exhaustion** was measured with Watkins et al.’s (2015) 3-item scale. A sample item is “I feel emotionally drained from my work.” The coefficient alpha was 0.869 for this survey.

We measured perceived **psychological safety** using the Edmondson’s (1999) 7-item scale. A sample item is “It is safe to take a risk in this company.” The coefficient alpha was 0.794 for this survey.

**Organizational embeddedness** was estimated using the 3-item scale developed by Cunningham et al. (2005). A sample item is “I feel a strong link to my organization.” The coefficient alpha was 0.798 for this survey.

We controlled for gender, age, and company tenure. In addition, because research was conducted in the context of Chinese culture, zhongyong was set as a control variable. Zhongyong is a core characteristic in Confucian culture. This factor was important, because research has shown that east Asians generally behave in ways compatible with the “zhongyong-oriented action model” (Yao et al., 2010; Chou et al., 2014). We assessed **zhongyong** using a 6-item scale (Du et al., 2014). A sample item is “Everything has limitations, so it is not very good to exceed them.” The coefficient alpha was 0.803 for this survey.

## Results

### Confirmatory Factor Analysis

We conducted confirmatory factor analyses using Amos 23.0 to confirm the construct validity. Table 1 shows the five-factor model provided a better fit (*χ*^2^ = 797.614, *df* = 420, CFI = 0.894, TLI = 0.882, RMSEA = 0.063) compared to the four-factor (*χ*^2^ = 880.858, *df* = 424, CFI = 0.871, TLI = 0.859, RMSEA = 0.069) and single-factor (*χ*^2^ = 2439.014, *df* = 434, CFI = 0.436, TLI = 0.395, RMSEA = 0.143) models. This corroborated the distinctiveness of the five measures.

**Table 1:** Comparison of measurement models.

Models	*χ*^2^	*df*	*χ*^2^/*df*	RMSEA	CFI	IFI	TLI
Five-factor model	797.614	420	1.899	0.063	0.894	0.895	0.882
Four-factor model	880.858	424	2.077	0.069	0.871	0.873	0.859
Single-factor model	2439.014	434	5.620	0.143	0.436	0.440	0.395

*N = 226. The five-factor model is the basic hypothesized measurement model. In the four-factor model, psychological safety and organizational embeddedness were combined. Finally, all items were combined into one factor to form a single-factor model*.

### Descriptive Statistics

Table 2 presents the means, standard deviations, correlations, and Cronbach’s alpha for the measures. The table shows that the Cronbach’s alpha for all measures was satisfactory (> 0.7). The table shows that psychological empowerment was positively correlated with psychological safety (*r* = 0.451, *p* < 0.01) and organizational embeddedness (*r* = 0.559, *p* < 0.01). Psychological empowerment was negatively related to emotional exhaustion (*r* = −0.370, *p* < 0.01). Moreover, both psychological safety (*r* = −0.406, *p* < 0.01) and organizational embeddedness (*r* = −0.485, *p* < 0.01) are negatively correlated with emotional exhaustion.

**Table 2:** Means, standard deviations, correlations, and Cronbach’s alpha.

S. No.	Variables	*M*	*SD*	1	2	3	4	5	6	7	8
1.	Gender	1.473	0.500	–							
2.	Age	32.195	5.220	−0.260^**^	–						
3.	Company tenure	6.089	4.959	−0.088	0.634^**^	–					
4.	Zhongyong	4.089	0.467	0.067	0.056	0.125	(0.803)				
5.	Psychological empowerment	3.679	0.520	−0.113	0.265^**^	0.148^*^	0.335^**^	(0.853)			
6.	Psychological safety	3.317	0.633	−0.108	0.090	0.103	0.207^**^	0.451^**^	(0.794)		
7.	Organizational embeddedness	3.478	0.824	−0.037	0.188^**^	0.120	0.190^**^	0.559^**^	0.538^**^	(0.798)	
8.	Emotional exhaustion	2.457	0.905	0.024	−0.055	−0.037	−0.096	−0.370^**^	−0.406^**^	−0.485^**^	(0.869)

*N* = 226. Values on the diagonal represent Cronbach’s alpha(*α*). Gender: 1 = male and 2 = female.

* *p* < 0.05;

** *p* < 0.01.

### Hypotheses Testing

Three steps were followed to test the hypotheses, using procedures developed by Baron and Kenny (1986). Firstly, the effect of psychological empowerment on emotional exhaustion was tested. Model 3 in Table 3 shows that psychological empowerment was negatively associated with emotional exhaustion (*B* = −0.689, *SE* = 0.120, *p* < 0.01). This result supported Hypothesis 1.

**Table 3:** Results of mediated regression analyses.

	Psychological safety	Organizational embeddedness	Emotional exhaustion	
	Model 1	Model 2	Model 3	Model 4
**Controls**				
Gender	−0.104(0.079)	0.062(0.096)	−0.019(0.118)	−0.024(0.110)
Age	−0.013(0.010)	0.007(0.012)	0.010(0.015)	0.009(0.014)
Company tenure	0.012(0.010)	0.002(0.012)	−0.004(0.015)	0.000(0.014)
Zhongyong	0.083(0.087)	−0.002(0.106)	0.071(0.130)	0.092(0.121)
**Independent Variable**				
Psychological empowerment	0.530^**^(0.080)	0.871^**^(0.098)	−0.689^**^(0.120)	−0.233(0.132)
**Mediators**				
Psychological safety				−0.263(0.101)^*^
Organizational embeddedness				−0.363(0.083)^**^
*R*^2^	0.218	0.316	0.375	0.526
*F*	12.238^**^	20.335^**^	7.179^**^	11.897^**^

*N* = 226. The coefficients reported in the models are all non-standardized coefficients.

* *p* < 0.05;

** *p* < 0.01.

Secondly, we examined the impact of psychological empowerment on psychological safety and organizational embeddedness. Model 1 and Model 2 in Table 3 present that psychological empowerment was positively related to psychological safety (*B* = 0.530, *SE* = 0.080, *p* < 0.01) and organizational embeddedness (*B* = 0.871, *SE* = 0.098, *p* < 0.01), respectively.

Thirdly, we verified the effect that psychological safety and organizational embeddedness on emotional exhaustion. As Model 4 in Table 3 suggests psychological safety (*B* = −0.263, *SE* = 0.101, *p* < 0.05) and organizational embeddedness (*B* = −0.363, *SE* = 0.083, *p* < 0.01) were significantly negatively correlated to emotional exhaustion. In contrast, psychological empowerment had no significant effect on emotional exhaustion (*B* = −0.233, *SE* = 0.132, *p* > 0.05). The results indicated that the effect of psychological empowerment on emotional exhaustion was mediated by psychological safety and organizational embeddedness. This supports Hypothesis 2 and Hypothesis 3.

Moreover, we applied the PROCESS macro in SPSS testing the mediator effect, which was developed by Hayes and Preacher (2013). Specifically, a bootstrapping analysis (5,000 samples) found that psychological safety had a significant mediator effect on the relationship between psychological empowerment and emotional exhaustion [*B* = −0.140, 95% CI (−0.268, −0.026)]. Organizational embeddedness also significantly mediated the relationship between psychological empowerment and emotional exhaustion [*B* = −0.316, 95% CI (−0.503, −0.166)]. Combining the above results, Hypothesis 2 and Hypothesis 3 were supported.

## Discussion

The primary goal of this study was to further explore the internal mechanism of influence that psychological empowerment on emotional exhaustion. Conservation of resources theory was applied to assess the role of psychological safety and organizational embeddedness as mediators. Drawing from resource loss and gain spirals, we proposed and verified a parallel mediation model to explain the linkage between psychological empowerment and emotional exhaustion.

### Theoretical Implications

First, the paper provides new evidence of a negative relationship between psychological empowerment and emotional exhaustion, focusing on study subjects in China. Previous studies show that psychological empowerment is negatively related to burnout symptoms (Spreitzer, 1997; Laschinger et al., 2003, 2004; Hochwälder, 2007; Boudrias et al., 2010, 2012; ÇAVUŞ and Demir, 2010; O’Brien, 2011; Tian et al., 2015; Schermuly and Meyer, 2016; Liu et al., 2019; Gong et al., 2021). Consistent with previous studies, we focus on emotional exhaustion, the key component of burnout. The study detailed how psychological empowerment prevents emotional exhaustion through two resources (psychological safety and organizational embeddedness) based on COR theory. Prior studies have shown that there is a negative correlation between job resources and burnout symptoms, especially along the cynicism dimension (Bakker et al., 2014).

This study’s findings enrich the current literature. The results demonstrate that psychological empowerment, psychological safety, and organizational embeddedness act as important job resources to help reduce emotional exhaustion. This further confirms that job resources can prevent the generation of negative emotions and can buffer the burnout that can result from job demands (Bakker et al., 2014). Further, because emotional exhaustion is the first sign of burnout development (Alarcon et al., 2011; Rubio et al., 2015), these findings expand the work of previous studies in considering the job resources as predictive variable of burnout.

Second, this study highlights new insights into psychological safety’s mediator role. Several studies have explored psychological safety’s mediator role in relationships between antecedent, such as organizational context, team characteristics, and team leadership; as well as outcomes, including innovation, performance, learning, and improvement within or by a team (Edmondson and Lei, 2014), and few studies pay attention to psychological safety’s relations to burnout (Newman et al., 2017). However, our studies find that at the individual level, with a higher level of psychological safety, people are less likely to suffer from the loss of resources and thus, emotional exhaustion, which is a predictor of job performance. Therefore, our findings enrich the current literature and may encourage researchers to include psychological safety as mediating roles when they examine variables at the individual level about the predictors of job performance.

Third, this study provides evidence that organizational embeddedness acts as a key mediating resource, linking job performance and its predictors. Research to date has started to identify embeddedness’s complicated mediator and moderator role in impacting other antecedents of performance (Lee et al., 2014). Regarding the negative determinants of job performance, many previous studies have focused on emotional exhaustion (Kong and Ho, 2018). This research confirms that the enhanced sense of embedded in work acts as a positive resource, can promote the resource gain spirals, and generate more positive resources to address emotional exhaustion.

Finally, the study also contributes to COR theory. Hobfoll et al. (2018) found that expanding the outcomes of resource losses and gains is vital in understanding COR theory. They provided new ideas for applying the theory to test resource investment processes. Our findings support this point of view and propose that when employees perceive a higher sense of psychological empowerment through cycles of resource loss and gain spirals, employees can experience a higher sense of psychological safety and organizational embeddedness. These positive job resources help them save and build more resources to cope with the possibility of emotional exhaustion. Some prior studies have also used the resources loss/gain spiral to explain emotions and stress-related problems. Hobfoll and Lilly (1993) found that resource losses were strongly correlated with emotional distress; however, resource gains were associated with psychological distress only after resource losses were controlled. In contrast, this study found new evidence that resource gain and loss spirals can function simultaneously to reduce the occurrence of emotional exhaustion.

### Practical Implications

World Health Organization (2019) included burnout as an occupational phenomenon in the 11th Revision of the International Classification of Diseases. Considering that emotional exhaustion is the key dimension of burnout, no matter for organizational mangers and practitioners in developing or developed countries, this study has important practical implications.

First, when combining results from this and previous studies, the research demonstrates the important role of psychological empowerment in reducing emotional exhaustion. Organizations can adopt human resource management practices designed to optimize the employee-job fit and develop employees’ senses of meaning, competence, self-determination, and impact (Pfeffer and Jeffrey, 1999). This is because empowered employees are better accoutered to address future change initiatives (Boudrias et al., 2012). This can be done by changing the work context, including building a reasonable reward system to recognize personal contribution and promoting access to information and resources (Spreitzer, 1995; Hochwälder, 2007).

Second, the study found that psychological safety acts as one mediator between psychological empowerment and emotional exhaustion. This indicates that psychological safety is a proximal factor affecting emotional exhaustion. Improving psychological safety is an effective strategy to intervene emotional exhaustion. In practice, to mitigate the risk of interpersonal communication and promote cooperation, it is important to create an atmosphere of psychological safety, particularly in uncertain, complex, and interdependent situations. Congruent communications and deliberate interventions are also important approaches for building and maintaining psychological safety (Edmondson and Lei, 2014). In addition, leaders play a critical role in creating psychologically safe environments. Leadership behaviors, such as being approachable, listening attentively, asking facilitative questions, demonstrating perseverance, and motivating participants to take action, actively help develop conditions for psychological safety (Edmondson and Lei, 2014).

Moreover, World Health Organization (2020) report that due to the nature of the work and working environment of healthcare workers, emotional exhaustion occurs more commonly among them, especially since the outbreak of the COVID-19 pandemic. Except physical risks, the epidemic has also brought severe psychological pressure to healthcare workers who have been in a high-demand environment for a long time. The WHO report recommends establishing a “no-fault” and fair work culture through open communication, including establishing legal and administrative protection measures to avoid being punished for reporting adverse safety incidents, which can effectively increase the sense of psychological safety and reduce emotional exhaustion.

Third, like psychological safety, the results demonstrate that organizational embeddedness is also a mediator between psychological empowerment and emotional exhaustion. Enhancing organizational embeddedness may also be effective in reducing emotional exhaustion. To motivate employees to be more embedded, managers can provide resources, including an attractive benefits package, links with coworkers within the workplace, and strong alignment between employees and current jobs (Lee et al., 2014). Furthermore, managers and executives can offer supportive leadership to help foster a supportive culture, promote cooperation among organizational members, and deepen their links with each other (Singh, 2018).

### Limitations and Future Directions

Like all studies, this research had some limitations. One limitation is its cross-sectional design, which does not fully support causal inferences. Future studies could apply quasi-experimental or longitudinal approaches to verify results. In addition, using COR theory revealed the influence of psychological empowerment on emotional exhaustion and its mediating mechanism; however, we did not explore the boundary conditions. Future studies could consider contextual factors as moderators to further enrich and improve the current model. Finally, the study was conducted under the background of Chinese culture, and it is unclear if the results apply in other cultures. The model and results could be tested in different cultural contexts in the future. Despite these limitations, this study is valuable to the field because we advance a dual mediation model to explain how high psychological empowerment results in low emotional exhaustion.

## Conclusion

In conclusion, through a dual mediation model, this study proposes and confirms that psychological empowerment was negatively correlated with emotional exhaustion, while psychological safety and organizational embeddedness mediated their relationship. Through COR’s resources loss and gain spiral separately, psychological empowerment increases the level of psychological safety and organizational embeddedness, which then represent as important factors to inhibit emotional exhaustion. The study provides a systematic view of the negative effect that psychological empowerment has on emotional exhaustion. We hope that this study will inspire future researchers to further explore other potential mechanisms alleviating emotional exhaustion through the two spirals of COR simultaneously.

## Data Availability Statement

The raw data supporting the conclusions of this article will be made available by the authors, without undue reservation.

## Ethics Statement

Ethical review and approval were not required for the study on human participants in accordance with the local legislation and institutional requirements. Written informed consent for participation was not required for this study in accordance with the national legislation and the institutional requirements.

## Author Contributions

HZ planned, designed and executed the study, reviewed and revised the draft. JC analyzed the data and prepared the first draft. All authors read and approved the submitted version.

### Conflict of Interest

The authors declare that the research was conducted in the absence of any commercial or financial relationships that could be construed as a potential conflict of interest.
